# Development of a quantitative rapid diagnostic test for multibacillary leprosy using smart phone technology

**DOI:** 10.1186/1471-2334-13-497

**Published:** 2013-10-23

**Authors:** Ludimila Paula Vaz Cardoso, Ronaldo Ferreira Dias, Aline Araújo Freitas, Emerith Mayra Hungria, Regiane Morillas Oliveira, Marco Collovati, Steven G Reed, Malcolm S Duthie, Mariane Martins Araújo Stefani

**Affiliations:** 1 Tropical Pathology and Public Health Institute, Federal University of Goiás, 235th Street, Setor Universitário, 74605-050 Goiânia-Goiás, Brazil; 2 Orange Life, Rio de Janeiro, Brazil; 3 Infectious Disease Research Institute, Seattle, USA

**Keywords:** Leprosy, Serologic response, Lateral flow, Brazil

## Abstract

**Background:**

Despite efforts to eliminate leprosy as public health problem, delayed diagnosis and disabilities still occur in many countries. Leprosy diagnosis remains based on clinical manifestations and the number of clinicians with expertise in leprosy diagnosis is in decline. We have developed a new immunochromatographic test with the goal of producing a simple and rapid system that can be used, with a minimal amount of training, to provide an objective and consistent diagnosis of multibacillary leprosy.

**Methods:**

The test immobilizes two antigens that have been recognized as excellent candidates for serologic diagnosis (the PGL-I mimetic, ND-O, and LID-1), on a nitrocellulose membrane. This allows the detection of specific IgM and IgG antibodies within 20 minutes of the addition of patient sera. Furthermore, we coupled the NDO-LID® rapid tests with a new cell phone-based test reader platform (Smart Reader®) to provide objective interpretation that was both quantifiable and consistent.

**Results:**

Direct comparison of serologic responses indicated that the rapid test detected a greater proportion of leprosy patients than a lab-based PGL-I ELISA. While positive responses were detected by PGL-I ELISA in 83.3% of multibacillary patients and 15.4% of paucibacillary patients, these numbers were increased to 87% and 21.2%, respectively, when a combination of the NDO-LID® test and Smart Reader® was used. Among multibacillary leprosy the sensitivity of NDO-LID® test assessed by Smart Reader® was 87% (95% CI, 79.2-92.7%) and the specificity was 96.1% (95% CI, 91.7- 98.6%). The positive predictive value and the negative predictive value of NDO-LID® tests were 94% (95% CI, 87.4-97.8%) and 91.4% (95% CI, 85.9-95.2%), respectively.

**Conclusion:**

The widespread provision of rapid diagnostic tests to facilitate the diagnosis or prognosis of multibacillary leprosy could impact on leprosy control programs by aiding early detection, directing appropriate treatment and potentially interrupting *Mycobacterium leprae* transmission.

## Background

Leprosy is a dermato-neurological disease that remains an important public health problem in many countries. Patients present with a spectrum of immunological, histological and clinical manifestations that depend on the host immune response to *Mycobacterium leprae*[1,2]. Clinical manifestations range from the polar lepromatous (LL) forms with high bacterial index (BI), weak *M. leprae* specific cell mediated immunity (CMI) and high antibody titers, to tuberculoid (TT) forms that have low BI, strong CMI and low antibody production. Early diagnosis and treatment are recognized as key elements in the prevention of long-term sequelae associated with leprosy, such as significant impairment of nerve function and deformities, and disabilities are found at greater frequency and severity in patients for whom diagnosis was significantly delayed [3]. In addition, multibacillary (MB) patients with high bacterial burdens are believed to be the primary transmitters of leprosy’s etiologic agent, *M. leprae*, so recognizing and treating infection in these individuals is likely to reduce transmission. Leprosy diagnosis is still, however, based on the recognition of clinical manifestations and reduction in the number of clinicians with expertise in its diagnosis, even in endemic countries, is likely contributing to delayed diagnosis or misdiagnosis [4,5].

There is an expectation that specific and sensitive tests will aid leprosy control programs to further the push toward eliminating this disease. With a goal of developing such tests, antibody responses of leprosy patients to *M. leprae* proteins and lipids have been extensively investigated, with *M. leprae*-specific phenolic glycolipid I (PGL-I) antigen representing the most thoroughly investigated to date. Several studies have demonstrated that the anti-PGL-I response is strong among MB patients and reflects the bacillary load, and lateral flow tests have previously been developed to detect the IgM response to PGL-I (ML Flow) and IgM, IgG and IgA antibodies (ML ICA) [6]. Not surprisingly given the limited antibody response of paucibacillary (PB) patients, these tests have limited sensitivity for the detection of PB patients with low BI [7,8]. No objective test for the diagnosis or prognosis of leprosy is, however, currently commercially available.

Our group has previously described novel antigenic proteins that clearly differentiate MB patients from healthy and *M. tuberculosis*-infected controls in endemic areas, therefore appearing suitable for the development of serologic tests for leprosy [9-11]. LID-1, a fusion of the ML0405 and ML2331 proteins, has proven to be strongly reactive with MB patients sera in many geographic locations [10,12-14]. Previous data have suggested that the addition of LID-1 protein to PGL-I could improve sensitivity in a diagnostic test, since some patients lacking anti-PGL-I antibodies have antibodies that recognize LID-1, and vice versa [12]. Furthermore, the progression of antibody responses against either PGL-I and LID-1 has been demonstrated to predict the onset of MB leprosy in experimental and clinical surveillance settings [10,14-16].

With a goal of producing a simple and rapid test that can be used with a minimal amount of training to provide an objective and consistent diagnosis of MB leprosy, we developed and evaluated a new rapid test for MB leprosy that incorporates both the LID-1 and PGL-I antigens. These antigens were immobilized on nitrocellulose membranes that permit transfer and detection of specific antibodies in patient’s sera. When coupled with a new cell phone-based test reader platform (Smart Reader® application), these tests can provide quantifiable and consistent data to assist in the diagnosis of MB leprosy. The development and widespread provision of rapid tests for MB leprosy diagnosis, prognosis or classification could have an important impact on leprosy control programs by facilitating/promoting early MB diagnosis, appropriate treatment and interruption of *M. leprae* transmission.

## Methods

### Study groups

Between 2006 and 2012, 441 participants were recruited in Goiânia city, Goiás State, Brazil, an endemic area for leprosy, under the approval of the local review board (Comitê de Ética em Pesquisa Humana e Animal/Hospital das Clínicas/Universidade Federal de Goiás) and National Ethics Commission (Comissão Nacional de Ética Pesquisa/CONEP/Brazil, protocols#4862/#12962). All participants (or legal guardians of patients under 18 years) signed an informed consent before blood collection. The test evaluations included five study groups: 1. Newly diagnosed, untreated MB leprosy patients (n = 108); 2. Newly diagnosed, untreated PB leprosy patients (n = 104); 3. Household contacts of MB and PB leprosy patients (HHC, n = 75); 4. Pulmonary tuberculosis patients with positive bacilloscopy, seronegative for HIV-1/2 and under specific treatment for at least three months (TB, n = 53); 5. Healthy endemic controls; defined as individuals without previous history of leprosy or TB diagnosis who were not intra-domicilary contacts of leprosy patients (EC, n = 101). Participants from both sexes and from all age ranges were included. Leprosy patients were recruited at the main regional public health outpatient clinic (Centro de Referência em Diagnóstico e Terapêutica, Goiânia city, Goiás State) and classified taking into consideration clinical, bacilloscopic and histopathologic data [1]. PB leprosy included TT and borderline-tuberculoid (BT) patients and MB group included patients in the borderline-borderline (BB), borderline-lepromatous (BL) and LL categories.

The Table 1 describes the main characteristics among the study groups. Among MB leprosy patients 36.1% were LL (39/108), 39.8% were BL (43/108) and 24.1% were BB (26/108), with a median bacterial index (BI) of 1.0. The PB leprosy group was composed by 41.4% TT patients (43/104) and 58.7% BT patients (61/104). The HHC group included both contacts of MB leprosy patients (80%; 60/75) and contacts of PB leprosy patients (20%; 15/75). The control groups included: TB patients (n = 53) and EC (n = 101).

**Table 1:** Main characteristics among the study groups recruited in Brazil

	**MB**	**PB**	**HHC**	**TB**	**EC**
**Sex (Female/Male)**	43/65	48/56	40/35	18/53	78/23
**Age (median range, years)**	48 (20–100)	42.5 (14–77)	36.5 (18–60)	40 (17–67)	25 (19–66)
**R & J classification**					
**LL**	39 (36.1%)	-	-	-	-
**BL**	43 (39.8%)	-	-	-	-
**BB**	26 (24.1%)	-	-	-	-
**BT**	-	61 (41.4%)	-	-	-
**TT**	-	43 (58.7%)	-	-	-
**Total/group**	108	104	75	53	101
**TOTAL =** 441					

*BB* borderline-borderline, *BL* borderline lepromatous, *BT* borderline- tuberculoid, *EC* healthy endemic controls, *HHC* household contacts, *LL* polar lepromatous, *MB* multibacillary leprosy, *PB* paucibacillary leprosy, *R & J* Ridley & Jopling, *TB* tuberculosis patients, *TT* polar tuberculoid.

### Enzyme linked immunosorbent assay (ELISA)

ELISA for the detection of IgM antibodies to PGL-I was performed as previously described [17,18]. In brief, the natural trisaccharide-phenyl analog of PGL-I conjugated to bovine serum albumin (NT-P-BSA) was used as antigen and the BSA alone was used as control (Sigma-Aldrich, St. Louis, USA). Serum samples were diluted 1:300 (PBS-BSA with 10% normal goat serum/NGS, Sigma-Aldrich, St. Louis, USA) and tested in duplicate. The reaction was developed by the addition of peroxidase-conjugated anti-human IgM (Cappel/Organon Teknika, Turnhout, Belgium) and 3,3′,5,5′-tetramethylbenzidine liquid as substrate system (TMB, Sigma-Aldrich, St. Louis, USA). To control intra- and inter-test variation, a standard reference serum was included in triplicate in each plate. The reaction was quenched by the addition of 2.5 N H_2_SO_4_ (Sigma-Aldrich, St. Louis, USA) when the optical density (OD) at 450 nm from the standard reference serum reached a value of 0.7. The cut-off value for positive results was OD ≥ 0.250. Total assay time was 3 hours.

### NDO-LID® rapid test

NDO-LID® rapid test (Orange Life®, Rio de Janeiro, Brazil) was developed by impregnating nitrocellulose membranes with ND-O-LID-1, a conjugation of the ND-O (a synthetic mimetic of PGL-I disaccharide) and the LID-1 protein [10,19,20]. Panels of well characterized sera from leprosy patients and controls were used to evaluate several prototypes in order to define the final test configuration. The NDO-LID® test is provided as a ready-to-use kit and was performed by adding undiluted serum (10 μl) and running buffer (100 μl) into the sample well, causing the migration of the sample and colloidal gold beads loaded with anti-IgG and anti-IgM through the membrane and across a detection window. Interactions with the test and/or control lines are revealed as a red color. Readings were performed after 20 minutes.

**Figure 1:**
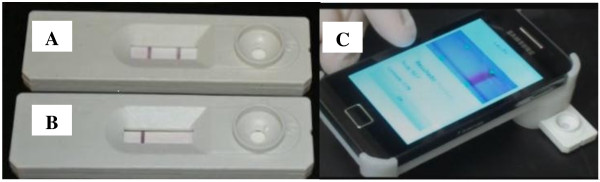
**NDO-LID® rapid test. A**.: A positive result was defined by the staining of both the control line and the test line (visual reading scores: 1+/1.5+/2+; Smart Reader® readings >10). **B**.: A negative result was defined when a clear control line, but only faint or absent test line, staining was observed. **C**.: Readings and objective scoring were achieved using a new Smart Reader® platform.

**Figure 2:**
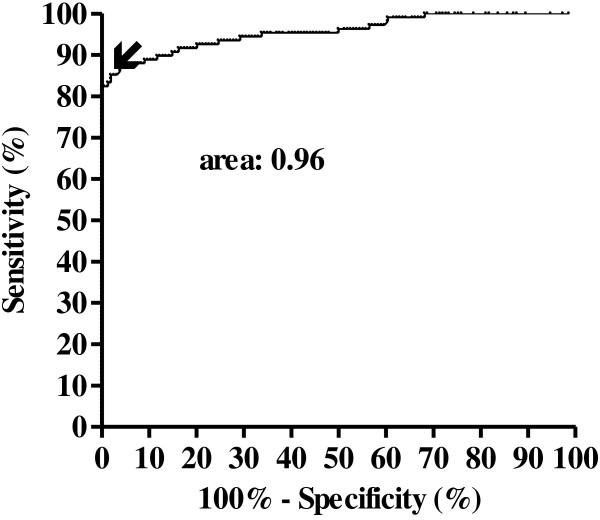
**Receiver Operating Curve (ROC) for NDO-LID® test for leprosy.** The insert indicates 87% of sensitivity.

Validation of the results required the visualization of a clear control line in the detection window. A positive result was defined by the staining of both the control line and the test line (visual reading scores: 1+/1.5+/2+); faint or no test line staining was considered as a negative result (Figure 1A and B). Visual readings were performed by three independent readers. In addition, readings were performed using a new Smart Reader® platform (Orange Life®) (Figure 1C) similar to that previously tested with malaria, TB and HIV rapid tests [21,22]. The calculation of Smart Reader® cut-off values was based on the Receiver Operating Curve (ROC), taking into account the visual results of the tests obtained with MB leprosy patient samples and control samples (TB and EC). Assuming a sensitivity of 87%, as determined by visual readings, the Smart Reader® cut-off was calculated as 9.99 (Figure 2). For data analysis the cut-off for positive results by Smart Reader® was therefore considered as 10.0. Assuming this cut-off, the sensitivity of the test for MB leprosy was 87% (95% confidence interval (CI): 79.2 to 92.7%) and the specificity was 96.1% (CI 95%: 91.7 to 98.6%), with an area under the curve (AUC) of 0.96 (standard deviation, sd 0.01; *p* < 0.0001). To validate readings performed by Smart Reader® for NDO-LID® test, the positive predictive value (PPV) and the negative predictive value (NPV) were calculated as 94% and 91.4%, respectively.

### Statistical analysis

Frequencies, median, mean of OD values and graphics were obtained (GraphPad Prism version 5). Statistical significance was assessed using Kruskal-Wallis one-way analysis of variance for comparison of multiple groups and by Mann–Whitney *U* test for comparison between two groups. Results were considered statistically significant when *p* values < 0.05 were obtained.

The concordance between results of the ELISA and NDO-LID® tests was determined by agreement and kappa values (κ) with 95% CI and sd value were calculated (Statistical Package for the Social Sciences, SPSS version 13.0). The following interpretation of agreement was used for the kappa values: low (0–0.5), moderate (0.51-0.75) and excellent (0.76-1.0) following established guidelines [23].

The accuracy of the NDO-LID® tests was evaluated by ROC (GraphPad Prism version 5) considering the AUC, in which a test that does not discriminate healthy individuals from patients, gives a value of AUC of 0.5 (null hypothesis). In our case the test discriminates MB leprosy from the other groups. A value above 0.7 is considered a satisfactory performance [24]. Moreover, the sensitivity, specificity and the PPV and NPV values were determined with 95% CI (GraphPad Prism version 5).

## Results

### Detection of MB patients by serologic tests

**Figure 3:**
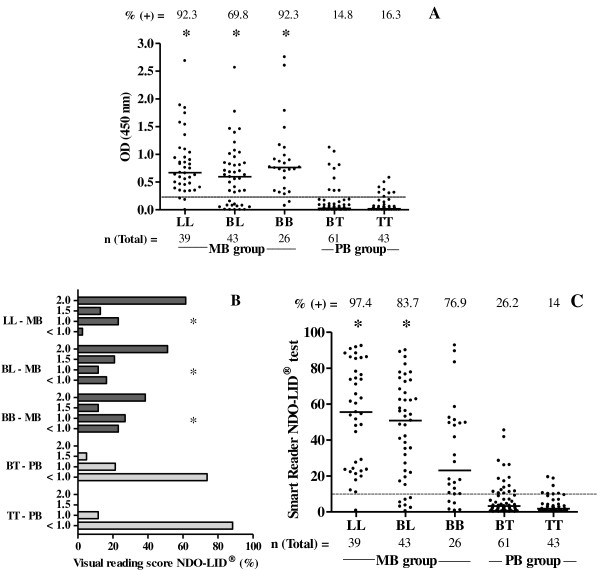
**PGL-I ELISA and NDO-LID® tests assessed among multibacillary (MB) and paucibacillary (PB) leprosy groups.** The number above each data set is the percentage of positive results and the number below each data set represents the total number of participants of each group. **A**.: IgM PGL-I ELISA test: each point represents the mean value of optical density (OD) of individual serum samples. The median OD value of each group is represented by the horizontal line. The traced horizontal line is the cut-off (OD ≥ 0.250). * = *p* < 0.05 (LL *versus* BT and TT; BL *versus* BT and TT; BB *versus* BT and TT). **B**.: Visual reading of NDO-LID® test scored * = *p* < 0.05. **C**.: NDO-LID® test assessed by Smart Reader® platform: each point represents the Smart Reader® result of an individual serum sample. The horizontal line represents the median of the Smart Reader® reading for each group and the traced horizontal line is the cut-off (≥ 10). * = *p* < 0.05 (LL *versus* BT and TT; BL *versus* BT and TT; BB *versus* BT and TT).

The anti-PGL-I ELISA positive rate among the MB leprosy patients tested was determined to be 83.3% (90/108; median OD = 0.766, range 0.285 to 2.755). As expected, seropositivity varied across the different MB leprosy categories: 92.3% for LL patients (36/39; median OD = 0.756, range 0.333 to 2.688), 69.8% for BL patients (30/43; median OD = 0.754, range 0.313 to 2.566) and 92.3% for BB patients (24/26; median OD = 0.772, range 0.285 to 2.755) (Figure 3A).

Among these same MB leprosy patients, the seropositive rate in NDO-LID® test was found to be slightly higher at 87% (94/108), with similar results obtained by both visual reading and the Smart Reader® platform (Figures 3B and C). These results demonstrate concordance at both performance and signal intensity of laboratory-based assays with the rapid test/Smart Reader® application. A decrease in the score of visual reading was observed across the spectrum of the disease from the MB (LL, BL and BB) to PB (TT, BT) pole when using the NDO-LID® tests (Figure 3B). Positive rates ranged from 97.4% for LL (38/39; median = 58, range 11 to 92.5), 83.7% among BL (36/43; median = 21.9, range 15.1 to 90.1) and 76.9% among BB leprosy (20/26; median = 44.9, range 10 to 44.9). Thus, similar declines in signal intensity across the clinical spectrum were measured by the Smart Reader® application, although a far greater range of signal intensity was captured by the electronic reader (Figure 3C). Although not statistically significant, the NDO-LID® rapid test actually slightly outperformed laboratory-based anti-PGL-I ELISA, suggesting an added benefit by including the LID-1 protein and indicating utility for the clear and simple detection of MB leprosy patients.

### Enhanced detection of PB leprosy by NDO-LID® test

As expected, when samples from PB leprosy patients were evaluated, the anti-PGL-I ELISA was found to be positive in only a minor subset, and with a reduced signal intensity compared to MB patients. An overall rate of 15.4% positive responders were found for PB patients (16/104; median OD = 0.454, range 0.300 to 1.126), ranging from 14.8% among BT patients (9/61; median OD = 0.359, range 0.346 to 1.126) to 16.3% for TT patients (7/43; median OD = 0.408, range 0.300 to 0.581) (Figure 3A).

As with the evaluations in MB patients, an increased number of PB patients was detected by the NDO-LID® test. The NDO-LID® rapid test was visually positive for 20.2% of PB leprosy patients (21/104): 26.2% for BT patients (16/61) and 11.6% for TT patients (5/43) (Figure 3B). NDO-LID® results assessed by Smart Reader® platform demonstrated a 21.2% positivity rate for PB leprosy patients (22/104): 26.2% among BT patients (16/61; median = 15.85, range 10.4 to 45.5) and 14% among TT patients (6/43; median = 12.6, range 10.2 to 19.5) (Figure 3C). Although these data indicate limited utility of the serological assays for detection of PB cases, they do indicate that some PB patients can actually be detected by this simple rapid test. This was particularly true for BT patients, where the rapid test almost doubled the detection rate over PGL-I ELISA.

### Seroreactivity among HHC and control groups

**Figure 4:**
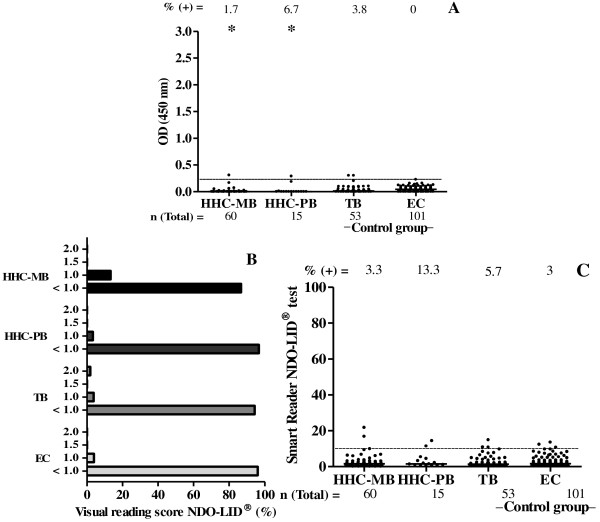
**PGL-I ELISA and NDO-LID® tests assessed among household contacts (HHC), tuberculosis (TB) patients and healthy endemic control (EC) groups.** The number above each data set is the percentage of positive results and the number below each data set represents the total number of participants of each group. **A**.: IgM PGL-I ELISA test: each point represents the mean value of optical density (OD) of individual serum samples. The median OD value of each group is represented by the horizontal line. The traced horizontal line is the cut-off (OD ≥ 0.250). * = *p* < 0.05 (HHC-MB *versus* TB and EC; HHC-PB *versus* EC). **B**.: Visual reading of NDO-LID® test scored. **C**.: NDO-LID® test assessed by Smart Reader® platform: each point represents the Smart Reader® result of an individual serum sample. The horizontal line represents the median of the Smart Reader® reading for each group and the traced horizontal line is the cut-off (≥ 10).

HHC of untreated MB patients represent an important study group as they are believed to be exposed to *M. leprae* at a higher frequency than the general population and seropositive contacts are considered to be at higher risk of progressing towards MB disease. In this study we tested HHC of both PB and MB patients. Overall, anti-PGL-I ELISA positivity among HHC was low (2.7%, 2/75): one contact from MB and another contact from PB leprosy patient (OD = 0.308 and 0.289, respectively) (Figure 4A).

For the NDO-LID® test an identical rate of seropositivity was detected in HHC by visual reading and by Smart Reader® (5.3%; 4/75); detecting 2 additional individuals along with both that were anti-PGL-I positive by ELISA (one of them HHC of MB patient and the other HHC of PB patient) (Figures 4B and C). For the two MB HHC seropositive, the Smart Reader® readings were 16.8 and 21.8 and for the two PB HHC seropositive, the Smart Reader® readings were 11.4 and 14.4, respectively.

Among TB patients the overall anti-PGL-I ELISA positivity was 3.8% (2/53; OD = 0.301 and 0.302) and no responses were observed among EC (Figure 4A). For TB patients tested by NDO-LID® the same positive rate was obtained by visual reading and by Smart Reader® readings (5.7%, 3/53). For EC 4% (4/101) were positive by visual reading, while 3% were positive using the Smart Reader® (3/101, *p* > 0.05) (Figures 4B and C). Together, these data indicate that, despite possible cross-reactivity with other mycobacteria circulating in the test populations, the rapid test is highly specific for leprosy.

### NDO-LID® rapid test enhances patient detection over anti-PGL-I ELISA

For the NDO-LID® test adopting the Smart Reader® cut-off ≥ 10, a high degree of agreement (99.1%; 437/441) was observed between the visual reading and the Smart Reader® (kappa value = 0.98; sd = 0.011) (Table 2). The four discordant cases were a single TT patient (negative by visual reading and Smart Reader® positive = 10.2), a single EC (positive by visual reading and Smart Reader® negative) and two TB patients (one positive by visual reading, negative in Smart Reader® and one negative by visual reading and Smart Reader® positive = 10.8).

**Table 2:** NDO-LID® test results: comparison between visual reading and Smart Reader®

**NDO-LID® test**		**Visual reading**		
		**Positive**	**Negative**	**Total**
**Smart Reader® platform**	**Positive**	124	3	127
	**Negative**	1	313	314
	**Total**	125	316	441

In 90.9% (401/441) of the samples anti-PGL-I ELISA and NDO-LID® results were concordant (either positive or negative; kappa value = 0.8; sd = 0.034) (Table 3). Among these, 98 were positive in both tests (86 MB leprosy; 10 PB; 2 HHC) and 303 samples were negative in both tests. The discordant rate observed between the NDO-LID® and anti-PGL-I ELISA test was 9.1% (40/441). A total of 28 NDO-LID® positive results were revealed among 331 anti-PGL-I ELISA negative samples: 8 from MB leprosy patients, 11 from PB leprosy, 2 from HHC, 3 from TB patients, 4 from EC. Conversely, only 12 negative NDO-LID® tests were found among anti-PGL-I positive samples: 4 from MB leprosy (median anti-PGL-I OD = 0.539, range 0.315 - 0.757), 6 from PB leprosy patients (median OD = 0.575, range 0.300 - 0.409) and 2 from TB patients (OD = 0.301 and 0.302).

**Table 3:** NDO-LID® and IgM anti-PGL-I ELISA results

		**NDO-LID® test**		
		**Positive**	**Negative**	**Total**
**PGL-I ELISA**	**Positive**	98	12	110
	**Negative**	28	303	331
	**Total**	128	313	441

## Discussion

Although leprosy remains an important public health problem in many countries, including Brazil, diagnosis is still based on the recognition of clinical manifestations by highly skilled clinicians [25]. A major concern in the widespread control efforts for leprosy is, however, that even in endemic regions the number of such clinicians that can actually recognize early signs or provide a confident differential diagnosis from other skin diseases is limited and in decline [4,26]. Tests that could be conducted on individuals with even a minimal suspicion of leprosy by general health workers, in conjunction with clinical exam by physicians, could facilitate referrals or initiate increased levels of monitoring.

The application of a rapid test for leprosy needs to take into account both the spectral nature of the disease and the immunological dichotomy in which at one pole PB patients develop weak (or absent) antibody responses while at the opposite pole MB patients develop a robust humoral immune response. The PGL-I and LID-1 antigens have been among the most reactive antigens tested in different populations, including multiple sites in Brazil, the Philippines, Japan, Nepal, Venezuela, Bangladesh, and India, suggesting utility in a global context [10,12,13]. As expected, the majority of MB leprosy patients were identified by serological assays, yielding higher responses than PB patients. Some studies have reported a moderate frequency of anti-PGL-I antibodies and anti-IgG antibodies to recombinant *M. leprae* proteins in PB leprosy patients (range 15 to 40%) [8,10,11]. An important limitation that must therefore be recognized for any serological test for leprosy is that their ability to detect PB patients may be limited. Given the results of anti-PGL-I ELISA, the seropositivity among PB leprosy patients by NDO-LID® rapid test in this study can be considered higher than expected (20.2%). The intra-domiciliary contacts of leprosy patients, especially MB patients, likely have a markedly increased rate of infection and absolutely have an increased risk of developing MB leprosy compared to the general population; among anti-PGL-I positive contacts this elevated risk is even higher [27,28]. We observed low response rates among contacts from both MB and PB leprosy patient groups, suggesting that positive tests may be indicative of symptomatic leprosy or asymptomatic *M. leprae* infection and not simply exposure. It should be noted that, even in a groups of 75 contacts and over 100 healthy controls as tested here, only few actual cases are predicted to emerge. Laboratory-based studies have already been used to alert surveillance teams to individuals that have developed clinical symptoms subsequent to strong results in LID-1 ELISA, at levels that are readily detected in the rapid test [14,16]. Expanded evaluations, either through bridging studies of archived sera or preferably through 'live’ testing as a component of sustained monitoring campaigns, are needed to better indicate the potential benefits of incorporating such a test by leprosy control programs in endemic countries. The rapidity and ease-of-use of the test also suggests that expanded screening within patient populations and their contacts including individuals younger than 15 years could be readily achievable and would provide important information for leprosy control programs.

For operational purposes a simplified leprosy classification system based on the number of skin lesions defines that PB leprosy presents with up to five lesions and usually include TT and BT forms whereas MB leprosy presents with more than five skin lesions and usually comprise LL, BL and BB forms [29]. However, the accuracy of this classification criterion is limited and may lead to misclassification and inadequate treatment [30,31]. Differentiation to the general classifications orients specific multidrug therapy (MDT) which consists of either six doses of rifampim and dapsone for PB leprosy or twelve doses of rifampim, dapsone and clofazimine for MB patients. Several studies have identified that anti-PGL-I responses and the ML Flow rapid test can help in the classification of MB and PB leprosy patients to orient the choice of treatment [6,8,32,33]. The rapid test that we have developed retains the ability to aid MB and PB leprosy classification, although the ability to 'diagnose’ PB leprosy remains limited. This is not surprising given the generally low anti-*M. leprae* antibody responses of PB patients. It has been previously reported that serum antibody responses to PGL-I and to LID-1 antigens decline upon MDT [15,34]. Importantly, given the enhanced fidelity of rapid test interpretation when incorporated with the Smart Reader®, monitoring of patients on treatment could potentially reveal complications such as relapses (or reinfection), providing improved patient care and management.

Typically, lateral flow-based rapid test have been scored subjectively on a grading system after visual inspection. Even with comparative examples, it is difficult to achieve consistency in these readings unless the reader(s) has garnered a large amount of experience. Additionaly, results from any diagnostic test would ideally be blinded from the clinical evaluation but in most settings, such as rural settings with limited resources and personnel or busy urban clinics, this is difficult to achieve. Considering the possibility of incorrect or bias in human reading of rapid tests in field conditions, an important innovation presented in this study is the use of a digital and automated rapid test Smart Reader® application. At the sole additional expense of a standard cell phone, this provides a means to generate controlled and consistent results across diverse endemic settings. As an extra layer of quality control and patient care, this compact pocket-size digital reader permits the transmission of digital data and other related information (e.g., demographic data) to a cloud, allowing the information to be downloaded for record keeping and/or be transferred to off-site experts for a rapid second opinion. This new technological strategy also allows real time spatio-temporal analysis of the disease prevalence and incidence in the region under evaluation.

For any serological test, both sensitivity and specificity represent the main parameters which were evaluated in this study by the inclusion of well characterized newly diagnosed, untreated leprosy patients (clinical, histopathological and microbiological features) and controls from an endemic area (EC and patients with TB). Among MB leprosy the sensitivity of NDO-LID® test assessed by Smart Reader® was 87% (95% CI, 79.2-92.7%) and the specificity was 96.1% (95% CI, 91.7- 98.6%). The PPV and the NPV of NDO-LID® tests were 94% (95% CI, 87.4-97.8%) and 91.4% (95% CI, 85.9-95.2%), respectively. Potential cross-reactivity is an important issue to be considered in the development of a new serologic test, particularly for countries with a high incidence of TB, high BCG vaccination coverage and high levels of exposure to environmental mycobacteria [35,36]. Using NDO-LID® test low positivity among healthy EC was observed. This is an important data since for most individuals from an endemic country, such as Brazil, there is a high chance of exposure to *M. leprae* throughout life especially in the highly endemic Northeast, North and Central Western regions, the latter representing the area where participants were recruited. Moreover the geographical region where participants were recruited is characterized by a very high BCG vaccination coverage (close to 100%). The low NDO-LID® positivity among TB and EC indicates that previous BCG vaccination, at least within the age range of participants is not associated with cross reactivity in the new rapid test [6,37]. The low seropositivity among TB patients observed with NDO-LID® test indicates that cross-reaction between *M. tuberculosis* antigens is not a significant concern. For the 3 (out of 53) TB patients with positive NDO-LID® test, subclinical infection or exposure to *M. leprae* cannot not be excluded. In general, the low positivity among control groups further supports the use of NDO-LID® test in an endemic country as Brazil.

## Conclusions

The new NDO-LID® rapid test presented in this study represents an important development in leprosy control, capable of providing a practical means to assist in the rapid, consistent and quantitative detection of MB leprosy. Considering the need for a rapid test in leprosy endemic countries, broader validation studies are being developed to evaluate performance under field conditions, including the direct use of finger prick blood samples to demonstrate its utility as a true point of care test. The NDO-LID® rapid test could ultimately aid leprosy control programs by allowing greater numbers of individuals to be simply tested at a greater frequency, and could be instrumental in the identification and prompt treatment of MB patients. Together, such applications of the test could help reach the WHO recommendation of early diagnosis to interrupt the transmission of *M. leprae* and further reduce leprosy case numbers.

## Abbreviations

AUC: Area under the curve; BI: Bacterial index; BB: Borderline-borderline; BL: Borderline-lepromatous; BT: Borderline-tuberculoid; BSA: Bovine serum albumin; CMI: Cell mediated immunity; CI: Confidence interval; ELISA: Enzyme linked immunosorbent assay; EC: Healthy endemic controls; HHC: Household contacts; κ: Kappa values; LL: Lepromatous; M. leprae: *Mycobacterium leprae*; M. tuberculosis: *Mycobacterium tuberculosis*; MB: Multibacillary; MDT: Multidrug therapy; NPV: Negative predictive value; NGS: Normal goat serum; OD: Optical density; PB: Paucibacillary; PGL-I: Phenolic glycolipid I; PPV: Positive predictive value; ROC: Receiver operating curve; sd: Standard deviation; SPSS: Statistical package for the social sciences; ND-O: Synthetic mimetic of PGL-I disaccharide; TT: Tuberculoid; TB: Tuberculosis; TMB: 3,3′,5,5′-tetramethylbenzidine.

## Competing interests

The authors declare that they have no competing interests. Marco Collovati is the owner of Orange Life® (Rio de Janeiro/Brazil), the company producing and marketing the NDO-LID® rapid test and Ronaldo Ferreira Dias, Orange Life® employee.

## Authors’ contributions

LPVC participated in the study designs, carried out the immunoassays, performed the statistical analysis and drafted the manuscript. RFD participated in the study design and carried out the immunoassays. AAF, EMH and RMO participated in the field work and carried out the immunoassays. MC participated in the study design. SGR participated in the study design and reviewed the manuscript. MSD conceived, participated in the study design and contributed to write the manuscript. MMAS conceived, designed the study, coordinated the field work, analyzed the data and contributed to write the manuscript. All authors read and approved the final manuscript.

## Pre-publication history

The pre-publication history for this paper can be accessed here:

http://www.biomedcentral.com/1471-2334/13/497/prepub
